# A revision of the tribe Planitorini van Achterberg (Hymenoptera, Braconidae, Euphorinae), with description of a new genus from Australia

**DOI:** 10.3897/zookeys.718.21151

**Published:** 2017-12-04

**Authors:** Cornelis van Achterberg, Donald L.J. Quicke, C. Andrew Boring

**Affiliations:** 1 Department of Terrestrial Zoology, Naturalis Biodiversity Center, Postbus 9517, 2300 RA Leiden, The Netherlands; 2 Integrative Ecology Laboratory, Department of Biology, Faculty of Science, Chulalongkorn University, Phayathai Road, Pathumwan, BKK 10330, Thailand; 3 9883 Versailles Southeastern Road, Versailles, Ohio 45380, U.S.A.

**Keywords:** Braconidae, Euphorinae, Planitorini, Mannokeraiini, *Paramannokeraia*, *Mannokeraia*, *Planitorus*, key, new genus, new species, distribution, Australia

## Abstract

The tribe Planitorini van Achterberg (Hymenoptera: Braconidae: Euphorinae) is revised. One new genus *Paramannokeraia* **gen. n.** (type species: *P. gibsoni* **sp. n.**) and five new species from Australia are described and illustrated: *Mannokeraia albipalpis* van Achterberg, **sp. n.**, *M. nigrita* van Achterberg, **sp. n.**, *M. punctata* van Achterberg, **sp. n.**, *Paramannokeraia gibsoni* van Achterberg & Quicke, **sp. n.** and *P. juliae* van Achterberg, **sp. n.** The tribe Mannokeraiini van Achterberg, 1995, is synonymized with the tribe Planitorini (**syn. n.**).

## Introduction

The subfamily Euphorinae Foerster, 1863 (Hymenoptera, Braconidae) is morphologically a very diverse group (Stigenberg et al. 2015), including many genera containing parasitoids of adult insects (Shaw and Huddleston 1991). The entirely Australian tribes Mannokeraiini van Achterberg, 1995, and Planitorini van Achterberg, 1995, are two aberrant groups, each containing only a single genus: *Mannokeraia* van Achterberg, 1995, with wingless females and *Planitorus* van Achterberg, 1995, with normally winged females (van Achterberg 1995). Accidently (partly because of their very derived morphology), the senior author referred the genera to the subfamilies Masoninae van Achterberg, 1995, and Betylobraconinae Tobias, 1979, respectively, but according to DNA analysis (Stigenberg et al. 2015) and some details of their morphology (e.g. the petiolate first metasomal tergite with its submedially situated spiracles) they belong to the subfamily Euphorinae. Earlier DNA analysis by Belshaw and Quicke (2002) and Sharanowski et al. (2011) corroborated already the inclusion of this group in the Euphorinae. In this paper the tribes Planitorini and Mannokeraiini are formally synonymised, and a new genus (*Paramannokeraia* gen. n.) and four new species are described and illustrated.

For the identification of the subfamily Euphorinae, see van Achterberg (1993), for more references see Yu et al. (2016) and for the terminology used in this paper, see van Achterberg (1979, 1988, 1993).

## Material and methods

The studied material concerns all the Planitorini and Mannokeraiini specimens used for DNA analysis and some additional specimens collected by Dr L. Masner (Ottawa). So far, the specimens have been provisionally identified up to genus level (Belshaw and Quicke 2002, Sharanowski et al. 2011, and Stigenberg et al. 2015). Observations and descriptions were made with an Olympus SZX11 stereomicroscope and fluorescent lamps. Photographic images were made with the Keyence VHX-5000 digital microscope and processed with Adobe Photoshop CS5, mostly to adjust the size and background.

Measurements are performed as indicated in van Achterberg (1988). The length of the first metasomal tergite is measured medially from apex of adductor muscle to apex of tergite. Additional non-exclusive characters in the key are between square brackets. The following abbreviations are used for the depositories: ANIC = Australian National Insect Collection, Canberra, Australia; CNC = Canadian National Collection of Insects, Ottawa, Canada; HIC = Hymenoptera Institute Collection, University of Kentucky, Lexington, USA; NHRS = Naturhistoriska Riksmuseet, Stockholm, Sweden.

## Taxonomy

### Planitorini

Taxon classificationAnimaliaHymenopteraBraconidae

van Achterberg, 1995

Figs 1 , 2–11 , 12–16 , 17–27 , 28 , 29–38 , 39–44 , 45 , 46–55 , 56–65 , 66 , 67–76 , 77–83 , 84–96

Planitorini van Achterberg, 1995: 46.
Mannokeraiini van Achterberg, 1995: 95. **Syn. n**.

#### Diagnosis.

Antenna of ♀ with 16–20 segments, and segments of apical half moniliform (Figs 11, 19, 38, 87), of ♂ with 28–32 segments and segments much longer than wide (Figs 13, 44, 51), pedicellus of ♀ narrower than scapus (Figs 19, 35, 58, 75; but much less so in *Planitorus*: Fig. 95); maxillary palp with 6 segments and labial palp with 4 segments; antennal sockets on facial protuberance, sockets remain separated from each other by distance from socket to eye (Figs 23, 35, 57) or touching each other (Fig. 85); mesosoma depressed (Figs 19, 87) or normal (Figs 3, 28, 58); scutellar sulcus wide and more or less curved (Figs 41, 62, 69) or narrow and curved (Figs 90); ♀ wingless (Fig. 18) or macropterous (Figs 2, 56, 84) as males; veins 3-M and 2-1A of fore wing largely unsclerotized (Figs 67, 84); vein m-cu of fore wing postfurcal (Figs 2, 56, 84); vein CU1b of fore wing absent (Figs 2, 29, 56, 84); vein 2-M of fore wing distinctly longer than vein 3-SR (Figs 2, 56, 84); vein M+CU of hind wing 2.0–2.5 times as long as vein 1-M and vein 1-M 1.3–2.0 times as long as vein 1r-m (Figs 2, 29, 56, 84); fore leg of ♀ robust (Figs 7, 21, 37, 63, 76, 89); first metasomal tergite narrow basally, more or less petiolate and its spiracle submedially situated (Figs 22, 32, 49, 59, 93), basal quarter or half of first metasomal tergite tube-shaped, first sternite more or less free from tergite in males of *Mannokeraia* and in other Planitorini, but ventrally closed in females of *Mannokeraia*.

#### Notes.

The DNA analysis by Stigenberg et al. (2015) clearly shows that despite the different general morphology of the adults both tribes belong together. The more or less developed facial prominence, the largely unsclerotized vein 3-M of fore wing, the basally narrow first tergite and the apical moniliform antennal segments of females are shared by all three genera.

#### Key to genera of the Planitorini van Achterberg

**Table:** 

1	Antennal sockets touching each other (Fig. 85); epistomal suture absent and clypeus not differentiated from face dorsally (Fig. 86); scutellar sulcus narrow and finely crenulate (Fig. 90); head elongate ventrally, malar space about 0.7 times height of eye in anterior view (Fig. 86); posterior half of mesopleuron depressed and divided into two parts by linear episternal scrobe (Fig. 87); fore tarsal segments of both sexes strongly widened (Fig. 94); face strongly convex medio-dorsally (Fig. 86); mesosternal sulcus absent and area smooth; postpectal carina absent medio-ventrally; pedicellus slightly narrower than scapus (Fig. 87)	***Planitorus* van Achterberg, 1995**
–	Antennal sockets remaining separate from each other (Figs 8, 35, 57); epistomal suture distinctly impressed and clypeus differentiated from face dorsally (Figs 8, 34, 52, 57); scutellar sulcus wide and coarsely crenulate (Figs 4, 62; less in wingless specimens: Fig. 18); head normal ventrally, malar space 0.15–0.30 times height of eye in anterior view (Figs 8, 23, 34, 57); posterior half of mesopleuron convex and undivided, only with elliptical episternal scrobe (Figs 30, 58); fore tarsal segments of ♂ slender and of ♀ moderately wide (Figs 37, 64); face moderately convex medio-dorsally (Figs 34, 57); mesosternal sulcus distinctly impressed and crenulate; postpectal carina variable, often present medio-ventrally; pedicellus much narrower than scapus (Figs 35, 58)	**2**
2	Dorsope of first tergite large and deep and tergite about 1.5 times longer than its apical width (Figs 59, 70, 78); ovipositor nearly cylindrical (Fig. 58); clypeus elliptical, medially high and ventrally flattened, without space between closed mandibles and clypeus (Figs 57, 73, 82); hind coxa at most basally finely rugose and remainder largely smooth (Figs 65, 66, 68); fore tibia of ♀ with at most some spiny bristles (Fig. 63, 76); both sexes macropterous	***Paramannokeraia* van Achterberg & Quicke, gen. n.**
–	Dorsope of first tergite absent or (rarely) shallowly impressed and tergite 2.2–2.9 times longer than its apical width (Figs 5, 22, 32, 49); ovipositor strongly compressed (Figs 1, 19, 20, 28); clypeus strongly transverse, medially low and with steep ventral face, and often with space between closed mandibles and clypeus (Figs 8, 23, 34); hind coxa completely rugose or punctate (Figs 27, 47); fore tibia of ♀ with distinct spines (Figs 7, 21, 37); ♀ wingless (Fig. 19) or both sexes macropterous	***Mannokeraia* van Achterberg, 1995**

### Mannokeraia

Taxon classificationAnimaliaHymenopteraBraconidae

van Achterberg, 1995

Figs 1 , 2–11 , 12–16 , 17–27 , 28 , 29–38 , 39–44 , 45 , 46–55

Mannokeraia van Achterberg, 1995: 96–97.

#### Type species.

*Mannokeraia aptera* van Achterberg, 1995 (examined).

#### Diagnosis.

**Figure 1.**
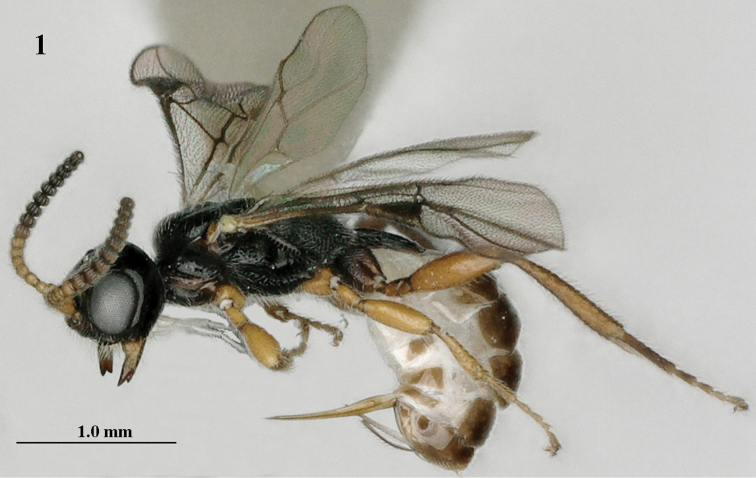
*Mannokeraia albipalpis* sp. n., ♀, holotype, habitus, lateral aspect.

Antenna of ♀ with 16–20 segments, and segments of apical half moniliform (Figs 11, 19, 38), of ♂ with 31–32 segments and segments much longer than wide (Figs 12, 44); clypeus strongly transverse and with steep ventral face, usually with transverse space between clypeus and closed mandibles (Figs 34, 52); absent in *M. aptera*: Fig. 23); head transverse and enlarged behind eyes (Figs 9, 17, 35, 43, 53); pronotal collar reaching level of mesoscutum in wingless females (Fig. 19), but much lower in macropterous specimens (Figs 3, 47); notauli reduced or united posteriorly (Figs 31, 41, 48); mesosternal sulcus distinct and crenulate; postpectal carina distinct medio-ventrally (but not visible in *M. aptera*); fore tibia of ♀ with distinct spines; hind coxa completely rugose; hind tibia densely striated; tarsal claws with a rounded lobe; dorsope of first tergite absent (at most weakly developed in *M. albipalpis*), 2.2–2.9 times longer than its apical width and tergite weakly widened posteriorly (Figs 5, 22, 32, 49); ovipositor strongly compressed (Figs 1, 19, 20, 28); ♀ wingless (Fig. 19) or both sexes macropterous (Figs 1, 28).

#### Distribution.

Australia: four species.

#### Biology.

Unknown.

#### Key to species of *Mannokeraia* van Achterberg

**Table:** 

1	Setose part of ovipositor sheath about 0.2 times as long as hind tibia (Fig. 19); propodeum smooth medially (Fig. 18); mesoscutum of ♀ at upper level of pronotum (Fig. 19); without space between clypeus and closed mandibles (Fig. 23); ♀ apterous (only with short wing pads: Fig. 18; ♂ unknown)	***M. aptera*** van Achterberg, 1995
–	Setose part of ovipositor sheath 0.5–0.6 times as long as hind tibia (Figs 1, 28); propodeum rugose or densely punctate medially (Figs 4, 31, 41, 49); mesosternum of both sexes far above upper level of pronotum (Figs 3, 30, 47); with transverse space between clypeus and closed mandibles (Figs 34, 52); both sexes macropterous	**2**
2	Propodeum densely and coarsely punctate (Figs 48, 49); pronotum and mesoscutum yellowish brown; [♀ unknown]	***M. punctata* sp. n.**
–	Propodeum only densely rugose (Figs 4, 31, 41); pronotum and mesoscutum dark brown or black	**3**
3	Basal 7 antennal segments of both sexes dark brown or blackish (Fig. 35); legs black; length of malar space equal to basal width of mandible (Fig. 34); palpi grey	***M. nigrita* sp. n.**
–	Basal 7 antennal segments of ♀ brownish yellow (Fig. 11), of ♂ scapus and pedicellus yellow and following 5 segments brown or dark brown; legs (except hind coxa) largely brownish yellow or yellowish brown; length of malar space 0.3 times basal width of mandible (Fig. 8); palpi white	***M. albipalpis* sp. n.**

### Mannokeraia albipalpis

Taxon classificationAnimaliaHymenopteraBraconidae

van Achterberg sp. n.

http://zoobank.org/54781D90-B529-41EE-9B8A-EE9CFAA9C787

Figs 1 , 2–11 , 12–16

Mannokeraia sp. 4 Stigenberg et al., 2015: 575.

#### Type material.

Holotype, ♀ (CNC), “**Austr[alia**], Qld., Mt. Glorious N.P., 630 m, 28.ii.1984, L. Masner s.s.”, “Wet rain forest”. Paratypes: 1 ♀ + 1 ♂ (HIC), “Australia: Qld., Main Range National Park, Nunningham’s Gap, Box Forest Track, elev. 700 m, yellow/blue/red pan traps (4:1:1) in creek bed, 400 m, S of parking area, 28°3.243'S 152°22.764'E, 10–11.xii.2005, A.R. Deans & M.L. Buffington”, “H4038” (only ♀), “DNA primary voucher AB 086 (♀) or AB 087 (♂), Hymenoptera Institute, University of Kentucky”.

#### Diagnosis.

Antenna of ♀ with 20 segments and medially slightly widened (Fig. 11), and basal 7 segments brownish yellow and apical 12 segments strongly moniliform, of ♂ scapus and pedicellus yellow and following 5 segments brown or dark brown; palpi white; with transverse space between clypeus and closed mandibles; head moderately enlarged behind eyes in dorsal view (Fig. 9); length of malar space 0.3 times basal width of mandible (Fig. 8); mesosoma of ♀ normal, with mesoscutum far above upper level of pronotum (Fig. 3); pronotum and mesoscutum black; propodeum rugose medially (Fig. 4); legs (except hind coxa) largely brownish yellow or yellowish brown; setose part of ovipositor sheath 0.5–0.6 times as long as hind tibia; both sexes macropterous.

**Figures 2–11.**
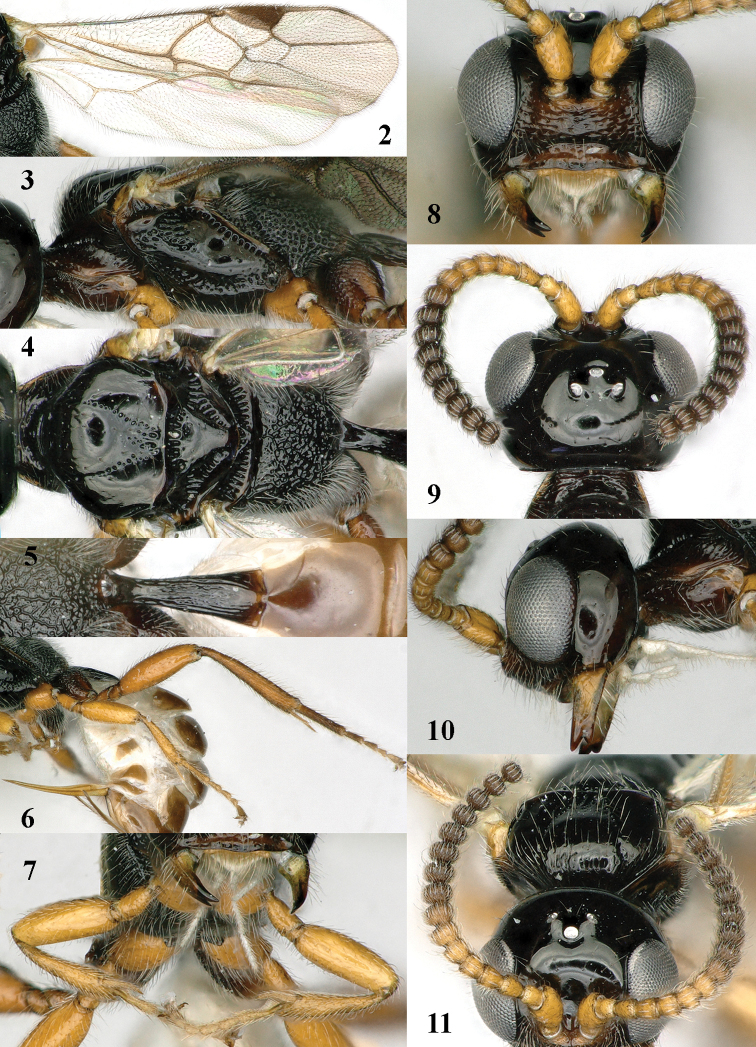
*Mannokeraia albipalpis* sp. n., ♀, holotype. **2** wings **3** mesosoma, lateral aspect **4** mesosoma, dorsal aspect **5** propodeum, first–third metasomal tergites, dorsal aspect **6** hind leg, lateral aspect **7** fore legs, inner aspect **8** head, anterior aspect **9** head, dorsal aspect **10** head, lateral aspect **11** antennae, ventral aspect.

#### Description.

Holotype, ♀, length of fore wing 2.9 mm, and of body 3.7 mm.

*Head.* Antenna with 16+ segments (apical segments missing, ♀ paratype has 20 antennal segments), length of third segment 1.1 times fourth segment, third and fourth segments 1.1 and 1.0 times as long as wide, respectively (Fig. 11) and with apical 9+ segments pedunculate, medially antenna wider than as apically; length of maxillary palp equal to height of head; occipital carina complete, comparatively low dorsally (Fig. 10), joining hypostomal carina below mandible and occipital flange elongate; eye 1.3 times as long as temple in dorsal view; temples subparallel-sided behind eyes; OOL:diameter of posterior ocellus:POL = 13:5:11; vertex and frons smooth and strongly shiny, with some long setae, convex, without median groove, and anteriorly flattened; face sparsely coarsely punctate and with some superficial rugae (Fig. 8); clypeus depressed and smooth ventrally, with ventral rim slightly upcurved, dorsally weakly convex and with some coarse punctures; length of malar space 0.3 times basal width of mandible; mandible flattened medially and with some striae, apically with large upper and medium-sized lower tooth (Fig. 10).

**Figures 12–16.**
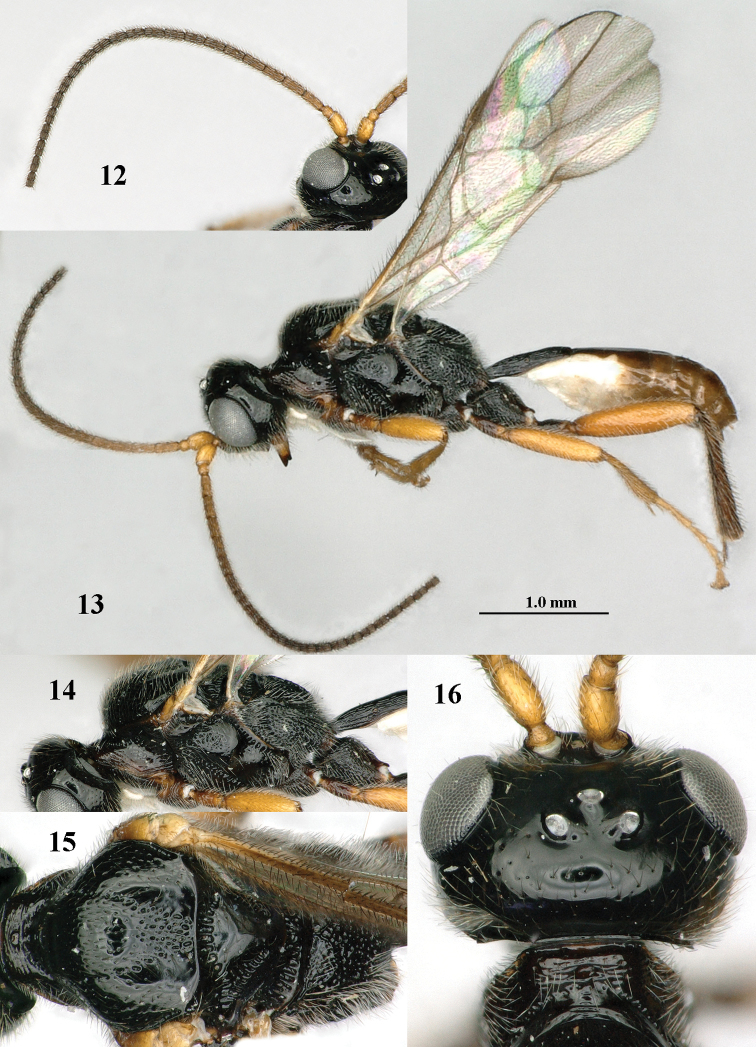
*Mannokeraia albipalpis* sp. n., ♂, paratype. **12** antenna, dorso-lateral aspect **13** habitus, lateral aspect **14** mesosoma, lateral aspect **15** mesosoma, dorsal aspect **16** head, dorsal aspect.

*Mesosoma.* Length of mesosoma 1.9 times its height; dorsal pronope and antescutal depression absent; side of pronotum narrowly crenulate antero-medially, widely crenulate postero-ventrally and remainder largely smooth; mesopleuron coarsely punctate dorsally; precoxal sulcus complete, rather widely crenulate-punctate (Fig. 3); remainder of mesopleuron smooth except for a few punctures; mesosternal suture rather deep and distinctly crenulate; postpectal carina distinct medio-ventrally; notauli complete and formed by narrow row of punctures; mesoscutum slightly convex, strongly shiny, and largely smooth, except for some coarse punctures medio-posteriorly (Fig. 4), glabrous laterally and with few long setae medially; scutellar sulcus with six costae; scutellum flat, smooth (except for some setiferous punctures) and shiny; metapleuron entirely coarsely punctate; propodeum entirely moderately reticulate-rugose (Figs 4, 5), its median carina absent, its posterior face rather differentiated and without tubercle postero-laterally (Fig. 4).

*Wings.* Fore wing: 1-M weakly curved; 1-SR short (Fig. 2); marginal cell closed anteriorly; 1-R1 1.7 times longer than pterostigma (Fig. 2); vein r emitted distinctly after middle of pterostigma; r:3-SR:SR1 = 4:16:87; vein SR1 straight; 2-SR:3-SR:r-m = 26:16:17; 2-M much longer than 3-SR; m-cu slightly postfurcal; 1-CU1 oblique and narrow, about as long as cu-a; 1-CU1:2-CU1 = 5:29; basal and subbasal cells of fore wing setose as other cells. Hind wing: marginal cell parallel-sided apically (Fig. 2); M+CU:1-M:1r-m = 28:14:10; basal and subbasal cells sparsely setose.

*Legs.* Hind coxa densely rugose-punctate, its outer side mainly punctate (Fig. 3); tarsal claws with wide truncate lamelliform lobe (Figs 6, 7); length of femur, tibia and basitarsus of hind leg 3.3, 6.4 and 6.2 times as long as their maximum width; fore femur inflated, 2.4 times longer than wide and apically rounded (Fig. 7); fore and middle tarsi slender (Figs 6, 7).

*Metasoma.* First tergite 2.5 times longer than its apical width, petiolate basally and gradually widened apically (Fig. 5), striate, dorsal carinae unite to form a median carina (Fig. 5), basal half of tergite closed ventrally and sternite differentiated; laterope absent; second tergite smooth; ovipositor sheath somewhat widened basally and obtuse apically (Fig. 1), its setose part 0.17 times as long as fore wing and 0.55 times hind tibia; ovipositor with minute subapical notch, compressed and basally widened (Fig. 6).

*Colour.* Black; palpi and basal half metasoma ventrally white; tegulae pale yellowish; seven basal segments of antenna, fore and middle legs brownish yellow; hind leg (except dark brown coxa) yellowish brown, but tibia and tarsus slightly darkened; face, clypeus, remainder of antenna and of metasoma (except black first tergite), pterostigma and most veins of fore wing dark brown; wing membrane weakly infuscate.

*Male*. Similar to female paratype except for the shape of the antennal segments (22+, apical segments missing; Fig. 12), slender fore femur and the different colour of base of antenna, legs and clypeus. Length of fore wing 3.3 mm, and of body 4.1 mm; antenna dark brown except for yellow scapus and pedicellus; clypeus brown ventrally; medio-posterior punctate area of mesoscutum rather large; coxae, trochanters, trochantelli, and hind tibia blackish or dark brown (hind tarsus missing); first tergite 2.9 times longer than wide apically, with dorsope shallowly impressed and distinctly longitudinally striate.

#### Variation.

Female paratype is very similar to holotype. Length of fore wing 2.9 mm, of body 3.0 mm; antenna with 20 segments, its penultimate segment as long as wide (without pedunculus 0.8 times); both teeth of mandible large; first metasomal tergite 2.3 times longer than its apical width and with slightly indicated dorsope; setose part of ovipositor sheath 0.18 times as long as fore wing and 0.54 times hind tibia.

#### Etymology.

Named after its white palpi (“albus” is white in Latin).

#### Distribution.

Australia (Queensland). Collected in December and February.

### Mannokeraia aptera

Taxon classificationAnimaliaHymenopteraBraconidae

van Achterberg, 1995

Figs 17–27

Mannokeraia apterus van Achterberg, 1995: 96-97, 153 (examined).

#### Diagnosis.

**Figures 17–27.**
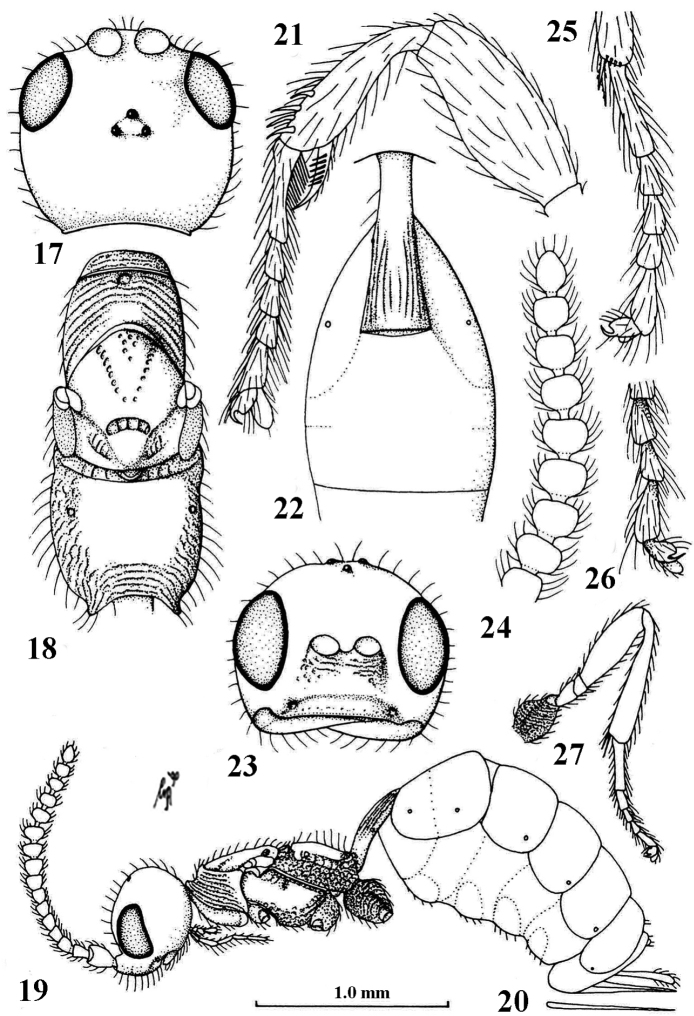
*Mannokeraia aptera* van Achterberg, ♀, holotype. **17** head, dorsal aspect **18** mesosoma, dorsal aspect **19** habitus, lateral aspect **20** ovipositor, ventral aspect **21**, fore tarsus, lateral aspect **22** first-third metasomal tergites, dorsal aspect **23** head, anterior aspect **24** apex of antenna, lateral aspect **25** middle tarsus, lateral aspect **26** outer hind claw, lateral aspect **27** hind leg, lateral aspect. 17, 18, 22, 23: 2.2× scale-line; 19, 20, 27: 1.0×; 21, 24, 25: 3.3×; 26: 2.5×. From: van Achterberg (1995).

Antenna of ♀ with 16 segments and medially widened (Fig. 19), basal 7 segments brownish yellow and apical 8 segments strongly moniliform; without space between clypeus and closed mandibles (Fig. 23); palpi pale yellowish; head strongly enlarged behind eyes in dorsal view (Fig. 17); length of malar space 0.8 times basal width of mandible; mesosoma of ♀ strongly depressed, with mesoscutum at upper level of pronotum (Fig. 19); pronotum and mesoscutum brown; propodeum smooth medially (Fig. 18); legs brownish yellow, except dark brown hind coxa and basitarsus; setose part of ovipositor sheath about 0.2 times as long as hind tibia; ♀ apterous (Figs 18, 19; ♂ unknown).

#### Distribution.

Australia (New South Wales, A.C.T.).

### Mannokeraia nigrita

Taxon classificationAnimaliaHymenopteraBraconidae

van Achterberg sp. n.

http://zoobank.org/8ABDB811-A37A-4E6A-A333-2BF7768FAD70

Figs 28 , 29–38 , 39–44

Mannokeraia sp. 1–3 Saranowski et al., 2011: 555, 559.

#### Type material.

Holotype, ♀ (ANIC), “**Australia**: Victoria, Bendae-Bonan, SE: Bonang Hwy, 56 km NNE Orbos, MT. in tree ferns in gully, 11.i.–12.ii.2005, 135 m, bulk no. 2619, 34°15’42"S 148°43’49"E, C. Lambkin, N. Starick, ANIC”, “DNA Voucher # BJS104, Hymenoptera Institute, University of Kentucky”. Paratypes: 3 ♂ (ANIC), same label data, but voucher numbers # BJS100, BJS100S and BJS105.

**Figure 28.**
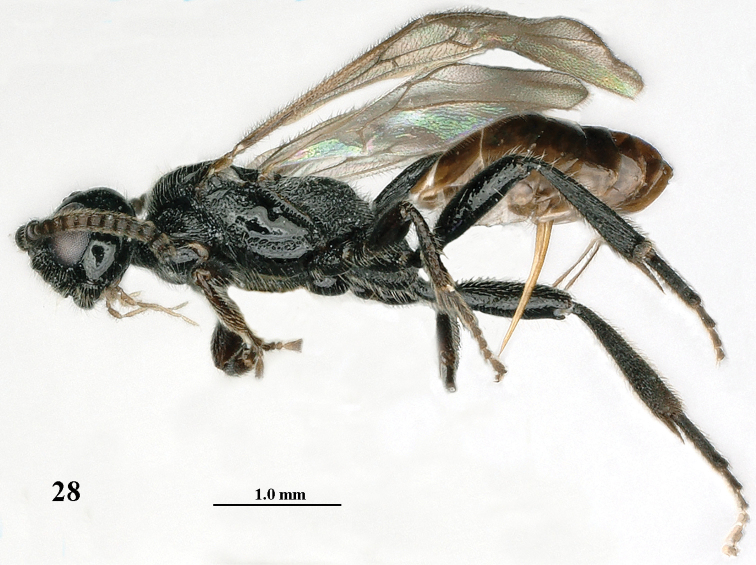
*Mannokeraia nigrita* sp. n., ♀, holotype, habitus, lateral aspect.

#### Diagnosis.

Antenna of ♀ with 19+ segments and medially rather widened (Fig. 38), and basal 7 segments dark brown and apical 12 segments strongly moniliform, of ♂ blackish (Fig. 44); palpi grey; with transverse space between clypeus and closed mandibles; head moderately enlarged behind eyes in dorsal view (Figs 35, 43); length of malar space equal to basal width of mandible (Fig. 34); mesosoma of ♀ normal, with mesoscutum far above upper level of pronotum (Fig. 30); pronotum and mesoscutum black; propodeum rugose medially (Fig. 31); legs blackish or dark brown; setose part of ovipositor sheath 0.7 times as long as hind tibia; both sexes macropterous.

**Figures 29–38.**
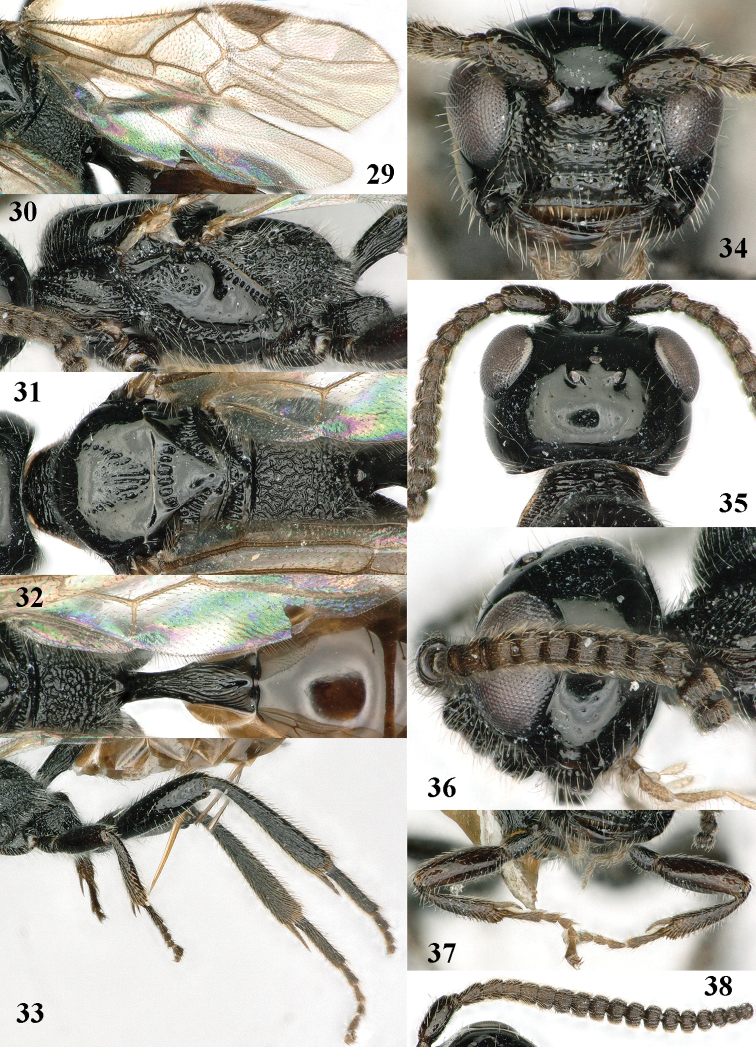
*Mannokeraia nigrita* sp. n., ♀, holotype. **29** wings **30** mesosoma, lateral aspect **31** mesosoma, dorsal aspect **32** propodeum, first–third metasomal tergites, dorsal aspect **33** hind leg, lateral aspect **34** head, anterior aspect **35** head, dorsal aspect **36** head, lateral aspect **37** fore legs, inner aspect **38** antenna, lateral aspect.

#### Description.

Holotype, ♀, length of fore wing 3.6 mm, and of body 5.1 mm.

*Head.* Antenna with 19+ segments (apical segment(s) missing), pedicellus short (Figs 35, 38), length of third segment 1.3 times fourth segment, third and fourth segments 1.3 and 1.0 times as long as wide, respectively (Fig. 38) and with apical 9+ segments pedunculate, medially antenna slightly wider than subbasally and apically distinctly narrowed (Fig. 38); length of maxillary palp 0.9 times height of head; occipital carina complete, comparatively low dorsally (Fig. 36), strongly curved ventrally and joining hypostomal carina below mandible and occipital flange curved and elongate; eye 1.1 times as long as temple in dorsal view; temples subparallel-sided behind eyes; OOL:diameter of posterior ocellus:POL = 14:5:15; vertex and frons smooth (but vertex with some punctures) and moderately shiny, with some long setae, convex, without median groove, and anteriorly flattened; face sparsely coarsely punctate and with some superficial rugae (Fig. 34); clypeus truncate (resulting in steep ventral face) and smooth ventrally, without ventral rim, dorsally weakly convex and with some coarse punctures; with wide transverse space between closed mandibles and clypeus; length of malar space equal to basal width of mandible; mandible flattened medially and coarsely striate, both apical teeth large.

**Figures 39–44.**
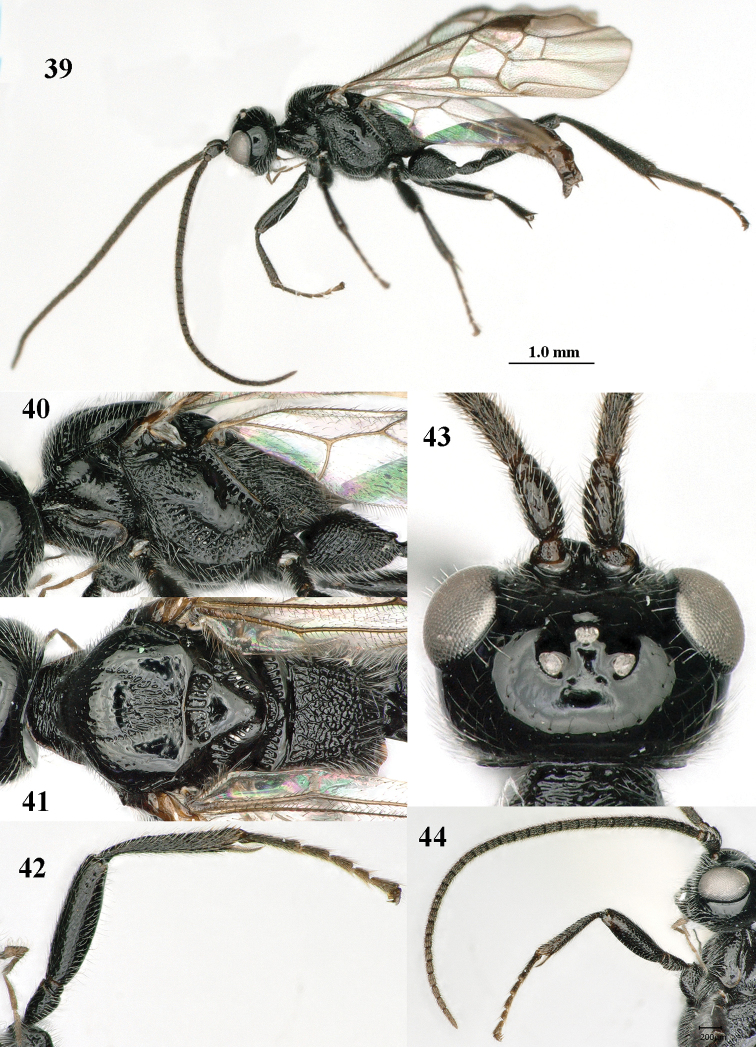
*Mannokeraia nigrita* sp. n., ♂, paratype. **39** habitus, lateral aspect **40** mesosoma, lateral aspect **41** mesosoma, dorsal aspect **42** fore leg, lateral aspect **43** head, dorsal aspect **44** antenna, lateral aspect.

*Mesosoma.* Length of mesosoma 1.9 times its height; dorsal pronope and antescutal depression absent; side of pronotum rugose, but medially and dorsally largely smooth; mesopleuron coarsely punctate dorsally; precoxal sulcus complete, rather narrow crenulate-punctate (Fig. 30); remainder of mesopleuron smooth except for a few punctures; mesosternal suture rather deep and coarsely crenulate; postpectal carina distinct medio-ventrally, straight; notauli complete, anteriorly a narrow row of punctures and posteriorly widely crenulate (Fig. 31); remainder of mesoscutum slightly convex, strongly shiny, and largely smooth, except for some coarse striae and punctures medio-posteriorly (Fig. 31), mesoscutum glabrous laterally and with few medium-sized setae medially; scutellar sulcus with five costae; scutellum flat, smooth (except for some setiferous punctures) and shiny; metapleuron entirely coarsely vermiculate-rugose; propodeum entirely moderately reticulate-rugose (Fig. 31), its median carina absent, its posterior face medially rather differentiated and without tubercle postero-laterally (Fig. 31).

*Wings.* Fore wing: pterostigma wide (Fig. 29); 1-M nearly straight; 1-SR short (Fig. 29); marginal cell closed anteriorly; 1-R1 1.5 times longer than pterostigma and direct after pterostigma hardly pigmented (as apex of pterostigma: Fig. 29); vein r emitted far after middle of pterostigma; r:3-SR:SR1 = 5:18:83; vein SR1 straight; 2-SR:3-SR:r-m = 28:18:19; 2-M much longer than 3-SR; m-cu slightly postfurcal; 1-CU1 oblique and narrow, about as long as cu-a; 1-CU1:2-CU1 = 5:31; basal and subbasal cells of fore wing similarly setose as other cells. Hind wing: marginal cell parallel-sided apically (Fig. 29); M+CU:1-M:1r-m = 32:15:10; basal and subbasal cells less densely setose than other cells.

*Legs.* Hind coxa largely rugose, dorso-basally transversely rugose; tarsal claws with wide truncate lamelliform lobe (Fig. 37); length of femur, tibia and basitarsus of hind leg 3.4, 5.5 and 5.2 times as long as their maximum width; fore femur inflated and ventrally flattened, 3.0 times longer than wide and apically rounded (Fig. 37); fore and middle tarsi rather flattened (Figs 33, 37); hind tibia distinctly striate.

*Metasoma.* First tergite 2.2 times longer than its apical width, petiolate basally and gradually widened apically (Fig. 32), coarsely striate but smooth posteriorly, dorsal carinae unite to form a median carina (Fig. 32), basal half of tergite closed ventrally and sternite differentiated; laterope absent; second tergite smooth; ovipositor sheath subparallel-sided and apically obtuse (Figs 28, 33), its setose part 0.26 times as long as fore wing and 0.73 times hind tibia; ovipositor with minute subapical notch, compressed and basally widened (Fig. 28).

*Colour.* Black; antenna and legs blackish or dark brown; palpi pale brown; tegulae, pterostigma (but apex pale), most veins of fore wing and metasoma (except black first tergite) dark brown; wing membrane weakly infuscate.

*Male*. Rather different (Fig. 39) from female holotype: clypeus more or less protruding medio-ventrally and with a weak rim ventrally, precoxal sulcus moderately to widely rugose (Fig. 40), distinctly curved postpectal carina, slender tarsi (Figs 39, 42, 44) and more pronounced sculpture of body. Length of fore wing 4.0–4.3 mm, and of body 4.3–4.6 mm; antenna with 31(2) or 32(1) segments; fore and middle tarsal segments slender; first tergite 2.2–2.3 times longer than wide apically and dorsope absent or slightly indicated.

#### Etymology.

Named after its blackish antenna (“niger” is black in Latin).

#### Distribution.

Australia (Victoria). Collected in January–February.

### Mannokeraia punctata

Taxon classificationAnimaliaHymenopteraBraconidae

van Achterberg sp. n.

http://zoobank.org/2C86782D-16FA-469A-9E4E-A3DE62FDCDEC

Figs 45 , 46–55

#### Type material.

Holotype, ♂ (CNC), “**Aust[ralia**]: Qld., Mt. Glorious N.P., 630 m, 28.ii.1984, L. Masner s.s.”.

**Figure 45.**
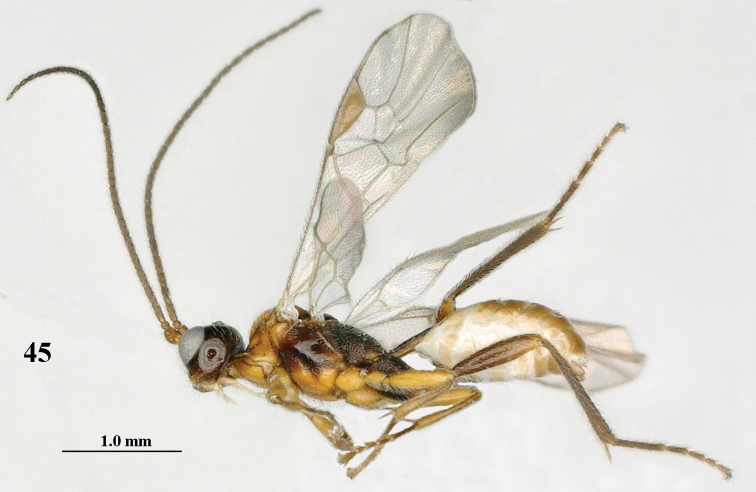
*Mannokeraia punctata* sp. n., ♂, holotype, habitus, lateral aspect.

#### Diagnosis.

Antenna of ♀ unknown, of ♂ with 30 segments, cylindrical and slender, dark brown but scapus and pedicellus brownish yellow ventrally; palpi pale yellowish; with transverse space between clypeus and closed mandibles (Fig. 52); head moderately enlarged behind eyes in dorsal view (Fig. 53); length of malar space 0.9 times basal width of mandible (Fig. 52); mesosoma of ♀ normal, with mesoscutum far above upper level of pronotum (Fig. 47); pronotum and mesoscutum yellowish brown; propodeum mainly punctate medially (Fig. 49); fore and middle legs (but tibiae and tarsi darkened) brownish yellow, and hind leg dark brown; length of setose part of ovipositor sheath unknown.

**Figures 46–55.**
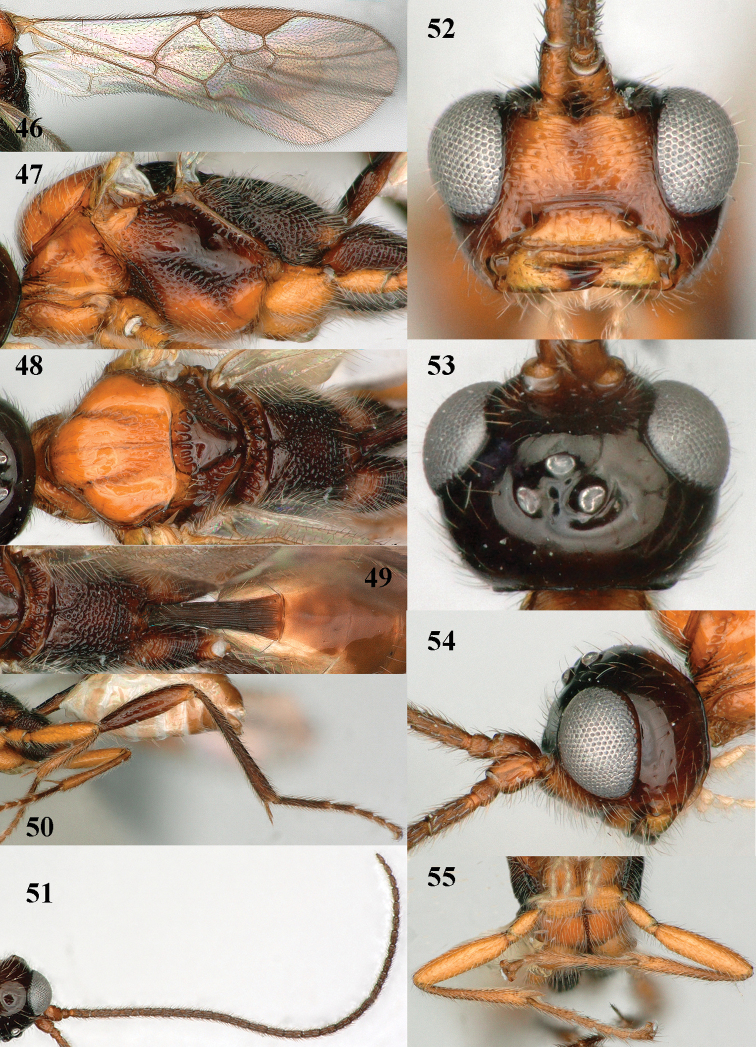
*Mannokeraia punctata* sp. n., ♂, holotype. **46** wings **47** mesosoma, lateral aspect **48** mesosoma, dorsal aspect **49** propodeum, first–third metasomal tergites, dorsal aspect **50** hind leg, lateral aspect **51** antenna, lateral aspect **52** head, anterior aspect **53** head, dorsal aspect **54** head, lateral aspect **55** fore legs, inner aspect.

#### Description.

Holotype, ♂, length of fore wing 2.8 mm, and of body 3.5 mm.

*Head.* Antenna with 30 segments, pedicellus short (Figs 51, 54), length of third segment 1.2 times fourth segment, third, fourth and penultimate segments 2.9, 2.4 and 2.7 times as long as wide, respectively (Fig. 51) and with apical segments sessile, medially parallel-sided and apically slightly narrowed (Fig. 51); length of maxillary palp 1.1 times height of head; occipital carina complete, comparatively low dorsally (Fig. 54), strongly curved ventrally and joining hypostomal carina far below mandible and occipital flange curved and elongate; eye 1.1 times as long as temple in dorsal view; temples slightly narrowed behind eyes; OOL:diameter of posterior ocellus:POL = 6:5:8; vertex and frons smooth (but vertex with some punctures) and moderately shiny, with some long setae, convex, frons without median groove, and anteriorly flattened; face sparsely coarsely punctate and with some superficial rugae (Fig. 52); clypeus flattened and smooth ventrally, with medially weakly protruding thick ventral rim (resulting in steep ventral area), dorsally weakly convex and with few coarse punctures; with medium-sized transverse space between closed mandibles and clypeus; length of malar space 0.9 times basal width of mandible; mandible slightly convex medially and with few punctures, both apical teeth large.

*Mesosoma.* Length of mesosoma 1.7 times its height; dorsal pronope and antescutal depression absent; side of pronotum antero-medially and posteriorly coarsely crenulate, rugose, antero-ventrally rugose and remainder largely smooth; mesopleuron coarsely punctate dorsally; precoxal sulcus complete, wide medially and coarsely punctate (Fig. 47); remainder of mesopleuron smooth; mesosternal suture rather narrow and finely crenulate; postpectal carina complete medio-ventrally, curved; notauli complete, anteriorly moderately crenulate and posteriorly ending in wide rugose area (Fig. 48); remainder of mesoscutum slightly convex, strongly shiny, and largely smooth, except for some fine punctures (Fig. 48), mesoscutum sparsely setose laterally and moderately setose medially; scutellar sulcus with six costae; scutellum slightly convex, smooth (except for some setiferous punctures) and shiny; metapleuron entirely coarsely punctate; propodeum coarsely punctate, only anteriorly partly sparsely punctate (Figs 48, 49), its median carina absent, its posterior face medially rather differentiated and without tubercle postero-laterally (Fig. 47).

*Wings.* Fore wing: pterostigma wide (Fig. 46); 1-M slightly curved; 1-SR short (Fig. 46); marginal cell closed anteriorly; 1-R1 1.3 times longer than pterostigma and direct after pterostigma weakly pigmented (as apex of pterostigma: Fig. 46); vein r emitted far after middle of pterostigma; r:3-SR:SR1 = 5:6:53; vein SR1 straight; 2-SR:3-SR:r-m = 19:6:11; 2-M much longer than 3-SR; m-cu interstitial; 1-CU1 oblique and narrow, about as long as cu-a; 1-CU1:2-CU1 = 1:8; basal and subbasal cells of fore wing similarly setose as other cells. Hind wing: marginal cell parallel-sided apically, but hardly visible; M+CU:1-M:1r-m = 27:13:10; basal and subbasal cells less densely setose than other cells.

*Legs.* Hind coxa largely transversely striate but basally punctate (Figs 49, 50); tarsal claws with wide truncate lamelliform lobe (Fig. 55); length of femur, tibia and basitarsus of hind leg 4.3, 8.4 and 6.8 times as long as their maximum width; fore femur moderately widened and ventrally convex, 4.0 times longer than wide and apically rounded (Fig. 55); fore and middle tarsi slender and subcylindrical (Figs 45, 55); hind tibia distinctly striate.

*Metasoma.* First tergite 2.8 times longer than its apical width, petiolate basally and gradually widened apically (Fig. 49), coarsely striate, dorsal carinae unite to form a median carina (Fig. 49), basal half of tergite closed ventrally and sternite distinctly differentiated; laterope absent; second tergite smooth.

*Colour.* Black; scapus and pedicellus ventrally, clypeus, mandible, fore and middle legs (but tibiae and tarsi darkened) brownish yellow; palpi and tegulae pale yellow; face, pronotum, mesoscutum, mesosternum and mesopleuron antero-dorsally and ventrally yellowish brown; remainder of antenna and of mesosoma, first metasomal tergite, hind leg, pterostigma (but apex pale), most veins of fore wing dark brown; remainder of metasoma brown, but ventrally membranes whitish; wing membrane subhyaline.

#### Etymology.

Named after its punctate propodeum (“punctus” is puncture in Latin).

#### Distribution.

Australia (Queensland). Collected in February.

### Paramannokeraia

Taxon classificationAnimaliaHymenopteraBraconidae

van Achterberg & Quicke gen. n.

http://zoobank.org/FC549892-3C5F-49AB-A922-319F9948553E

Figs 56–65 , 66 , 67–76 , 77–83

#### Type species.

*Paramannokeraia gibsoni* van Achterberg & Quicke, sp. n. Gender: feminine.

#### Etymology.

From “para” (= Greek for “near”) and the generic name *Mannokeraia* van Achterberg, 1995, because the new genus is related to it.

#### Diagnosis.

**Figures 56–65.**
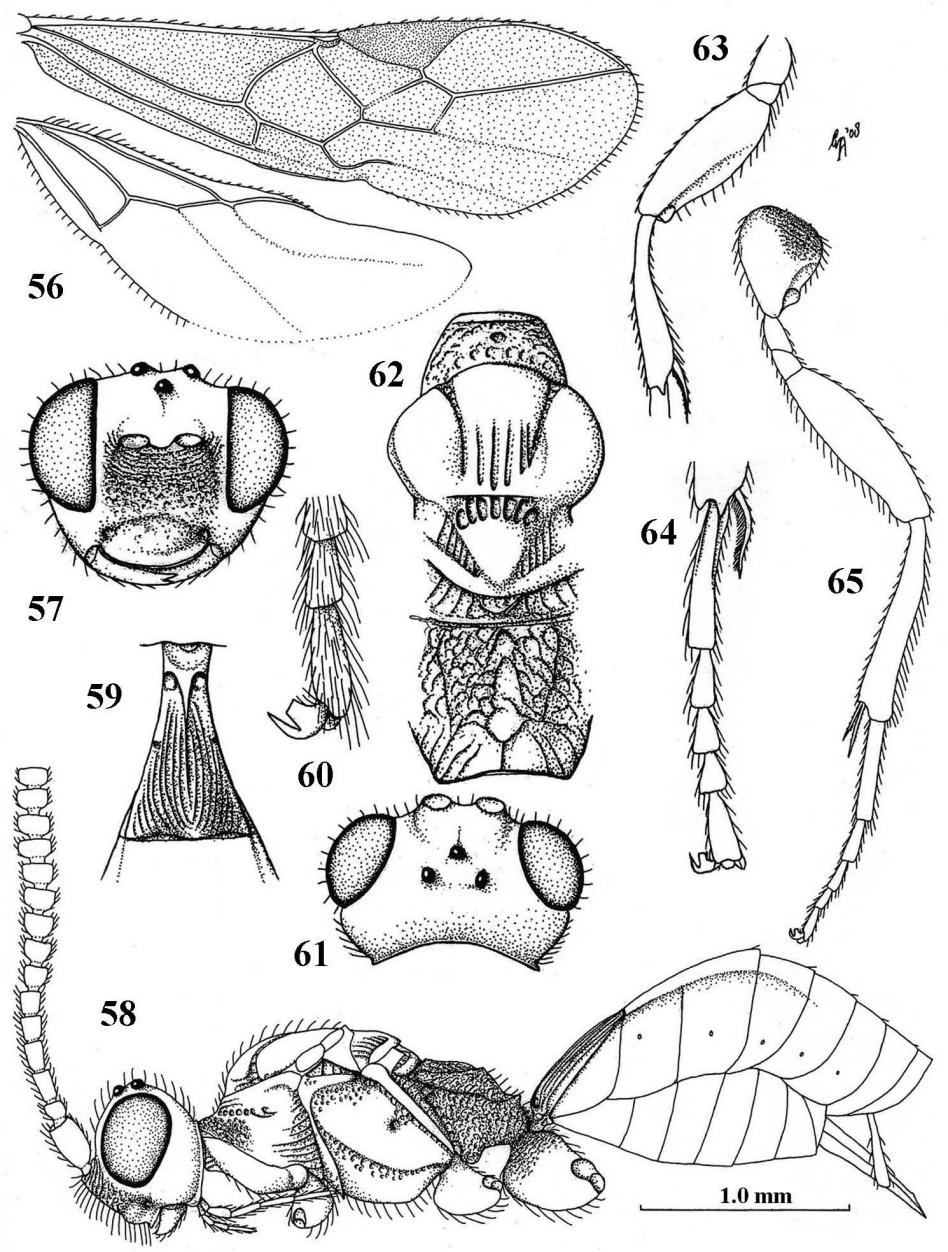
*Paramannokeraia gibsoni* gen. n. & sp. n., ♀, holotype. **56** wings **57** head, anterior aspect **58** habitus, lateral aspect **59** first metasomal tergite, dorsal aspect **60** outer hind claw, lateral aspect **61** head, dorsal aspect **62** mesosoma, dorsal aspect **63** fore femur and tibia, lateral aspect **64** fore tarsus, dorsal aspect **65** hind leg, lateral aspect. 56, 58, 65: scale-line (= 1×); 57, 59, 61, 62, 64: 2.0×; 63: 2.2×; 60: 3.2×.

Antenna of ♀ with 19 segments, pedicellus much narrower than scapus and most segments moniliform (Fig. 58), of ♂ with about 28 segments and segments much longer than wide; scapus much longer and wider than pedicellus (Fig. 58); face convex medio-dorsally (Fig. 58); maxillary palp with 6 segments and labial palp with 4 segments; eyes distinctly setose; clypeus rather large and elliptical (Fig. 57), dorsally differentiated from face and ventrally flattened; face moderately convex medio-dorsally (Fig. 57); pronotal collar long (Figs 58, 62) and distinctly below level of mesoscutum; notauli nearly complete (Fig. 64); scutellum without medio-posterior depression; mesosternal sulcus distinct and crenulate; postpectal carina variable (distinct in *P. gibsoni*, and absent, with at most the area between middle coxae rugose in *P. juliae*); vein M+CU1 of fore wing sclerotised; vein cu-a of hind wing present and comparatively close to vein 1r-m (Fig. 56); fore femur robust and flattened ventrally (Fig. 63); fore tibia without distinct spines and apically with wide tooth-like protuberance (Fig. 63); fore tibial spur medium-sized; base of fore basitarsus angulate (Fig. 64); telotarsi hardly widened (Figs 64, 65); hind tibia largely smooth between pimply protrusions; tarsal claws angularly bent and with truncate lobe (Fig. 60); propodeum without large posterior areola and median carina absent (Fig. 62), medio-posteriorly gradually lowered (Fig. 62); first tergite gradually widened posteriorly and with its spiracles submedially situated (Fig. 59) and tergite inserted near condyli of hind coxa; dorsope present (Fig. 59); laterope absent; ovipositor nearly cylindrical.

#### Distribution.

Australia (two species).

#### Notes.

Because of its venation, shape of the telotarsi, submedial position of the spiracle of the first tergite and shape of the first tergite, the genus belongs to the subfamily Euphorinae within which it belongs to the tribe Planitorini. It resembles *Mannokeraia*, because of the small pedicellus (much narrower than the scapus; Fig. 58), apical antennal segments of the female strongly moniliform and pedunculate, the face moderately convex medio-dorsally, the lack of the medio-posterior depression of the scutellum, the robust fore femur, the setose eyes and the long pronotal collar. According to the DNA analysis by Stigenberg et al. (2015) *Paramannokeraia* is sister to *Planitorus* (sharing the presence of dorsope on the first tergite, and the ventrally flattened and narrower clypeus); the two genera forming a sister group to *Mannokeraia* which has the first tergite lacking dorsope, and the clypeus transverse and with a steep ventral face.

#### Key to species of *Paramannokeraia* gen. n.

**Table:** 

1	Face rugose dorsally and densely punctate ventrally (Fig. 57); base of hind coxa finely rugose dorsally (Fig. 65); hind femur rather slender (Fig. 65); setose part of ovipositor sheath 0.2 times as long as hind tibia (Fig. 58); basal half of antenna and legs dark yellowish brown; apically fore tibia with tooth-like protuberance (Fig. 63); notauli on mesoscutal disk narrow (Fig. 62); fore and middle tarsi of ♀ slender (Fig. 64)	***P. gibsoni* sp. n.**
–	Face mainly sparsely punctate (Fig. 73); base of hind coxa smooth dorsally (Fig. 68); hind femur rather swollen (Fig. 71); setose part of ovipositor sheath 0.4 times as long as hind tibia (Fig. 66); basal half of antenna of both sexes and legs dark brown; fore tibia rounded apically, without tooth-like protuberance apically (Fig. 76); notauli on mesoscutal disk widened (Fig. 69); fore and middle tarsi of ♀ widened (Figs 66, 76)	***P. juliae* sp. n.**

### Paramannokeraia gibsoni

Taxon classificationAnimaliaHymenopteraBraconidae

van Achterberg & Quicke sp. n.

http://zoobank.org/232633BF-B8BA-4AEF-81BF-C3E2FA06C0F1

Figs 56–65

Mannokeraia gibsoni ; Belshaw and Quicke 2002: 474 (MS name for “Australia AJ416968”). Nomen nudum.

#### Type material.

Holotype, ♀ (CNC), “**Australia**: N.S.W., Mt. Keira via Wollongong, iv.2005”, “BF000332, RJF 004 D8”, “gen. n. aff. Planitorus, det. Belokobylskij, [20]08”.

#### Diagnosis.

Antenna of ♀ with 15+ robust segments, apical segments pedunculate (Fig. 58), of ♂ unknown; head transverse, not enlarged behind eyes in dorsal view (Fig. 61); face rugose dorsally and densely punctate ventrally (Fig. 57); mesosoma of ♀ normal, with mesoscutum above upper level of pronotum (Fig. 58); notauli on mesoscutal disk narrow (Fig. 62); propodeum rugose medially (Fig. 62); base of hind coxa finely rugose dorsally (Fig. 65); hind femur rather slender (Fig. 65); apically fore tibia with tooth-like protuberance (Fig. 62); fore and middle tarsi of ♀ slender (Fig. 64); basal half of antenna and legs rather dark yellowish brown; setose part of ovipositor sheath about 0.2 times as long as hind tibia; ♀ macropterous.

#### Description.

Holotype, ♀, length of fore wing 2.1 mm, and of body 2.2 mm.

*Head.* Antenna with 15+ segments, length of third segment 1.1 times fourth segment, third and fourth segments 1.7 and 1.6 times as long as wide, respectively (Fig. 58), apical segments pedunculate (Fig. 58); length of maxillary palp 1.1 times height of head; occipital carina complete, low dorsally (Fig. 58); eye 1.8 times as long as temple in dorsal view; temples gradually narrowed behind eyes (Fig. 61); OOL:diameter of posterior ocellus:POL = 6:3:9; frons smooth, with long setae and without median groove or carina, slightly depressed; face rather coarsely rugose dorsally and punctate ventrally (Fig. 57); clypeus depressed and smooth ventrally, dorsally weakly convex and with some punctures (Fig. 57); length of malar space 0.8 times basal width of mandible; occipital carina about joining hypostomal carina and occipital flange subcircular (Fig. 58); mandible flat and shiny basally.

*Mesosoma.* Length of mesosoma 1.8 times its height; dorsal pronope small, round (Fig. 62); antescutal depression absent; side of pronotum largely reticulate-punctate anteriorly, medially largely smooth and posteriorly punctate-costate (Fig. 58); epicnemial area coarsely punctate dorsally; precoxal sulcus complete, coarsely punctate (Fig. 58) and remainder of mesopleuron smooth; mesosternal suture rather deep and moderately crenulate; postpectal carina present medio-ventrally; mesoscutum flat, smooth (except five grooves medio-posteriorly: Fig. 62), glabrous laterally and with long setae medially; notauli nearly complete, largely smooth and narrow (Fig. 62); scutellar sulcus with five costae; scutellum flat, smooth (also medio-posteriorly: Fig. 62); metapleuron coarsely and densely rugose-punctate; propodeum coarsely and densely rugose but less so posteriorly, its median carina absent except posteriorly (Fig. 62), its posterior face weakly differentiated and with an obtuse tubercle postero-laterally, just above level of socket of first tergite (Fig. 58).

*Wings.* Fore wing: 1-M distinctly curved; 1-SR very short (Fig. 56); marginal cell closed anteriorly; vein r emitted distinctly after middle of pterostigma; r:3-SR:SR1 = 3:10:54; vein 1-R1 somewhat longer than pterostigma; vein SR1 straight; 2-SR:3-SR:r-m = 10:5:6; 2-M distinctly longer than 3-SR; m-cu postfurcal; 1-CU1 oblique and narrow; 1-CU1:2-CU1 = 7:20; basal and subbasal cells of fore wing setose as other cells. Hind wing: marginal cell subparallel medially and absent apically (Fig. 56); M+CU:1-M:1r-m = 38:15:12.

*Legs.* Hind coxa basally finely rugose and remainder largely smooth (Fig. 58); tarsal claws with wide truncate lamelliform lobe (Fig. 60); length of femur, tibia and basitarsus of hind leg 3.2, 6.2 and 6.0 times as long as their maximum width; fore femur rather inflated, 2.7 times longer than wide, with apical tooth and with some spiny bristles (Fig. 63).

*Metasoma.* First tergite 1.5 times longer than its apical width, distinctly petiolate (Fig. 59), with coarse curved striae, dorsal carinae unite to form a median carina and dorsope deep and large (Fig. 59), only basal quarter closed ventrally; laterope absent, tergite widened latero-basally (Fig. 58); second tergite smooth; ovipositor sheath somewhat widened and obtuse apically (Fig. 58), its setose part 0.10 times as long as fore wing and 0.23 times hind tibia; ovipositor with minute subapical nodus and wide basally (Fig. 58).

*Colour.* Black; basal half of antenna, pronotum narrowly antero-ventrally and legs rather dark yellowish brown; tegulae and palpi pale yellowish; metasoma (except black first tergite), pterostigma (but narrowly paler basally) and apical half of antenna dark brown; veins brown; wing membrane weakly infuscate.

#### Etymology.

Named after Dr Gary A.P. Gibson (Ottawa), for his extensive contribution to our knowledge of Chalcidoidea (especially of the families Eupelmidae and Pteromalidae), and of Mymarommatidae.

#### Distribution.

Australia (New South Wales). Collected in April.

### Paramannokeraia juliae

Taxon classificationAnimaliaHymenopteraBraconidae

van Achterberg sp. n.

http://zoobank.org/964AFC04-F505-4C18-ADC3-2F72062294D6

Figs 66 , 67–76 , 77–83

Mannokeraia sp.; Stigenberg et al. 2015: 575.

#### Type material.

Holotype, ♀ (NHRS), “**Australia**, **Tasmania**, Cradle Mtn NP, creek from Crater Lake to Ronny Creek, 100 m upstr. boardwalk, 867 mao, S41°38.667' E145°56.755', 23.ii–4.iii.2006, Malaise trap, loc. 14, N. Jönsson, T. Malm & D. Williams”, “DNA voucher DNA JS10_00282”, “NHRS-HEVA 000004017”. Paratype: 1 ♂ (CNC), “Australia, Tas[mania], Mt. Field NP, 7.i.1984, L. Masner, s. s.”.

**Figure 66.**
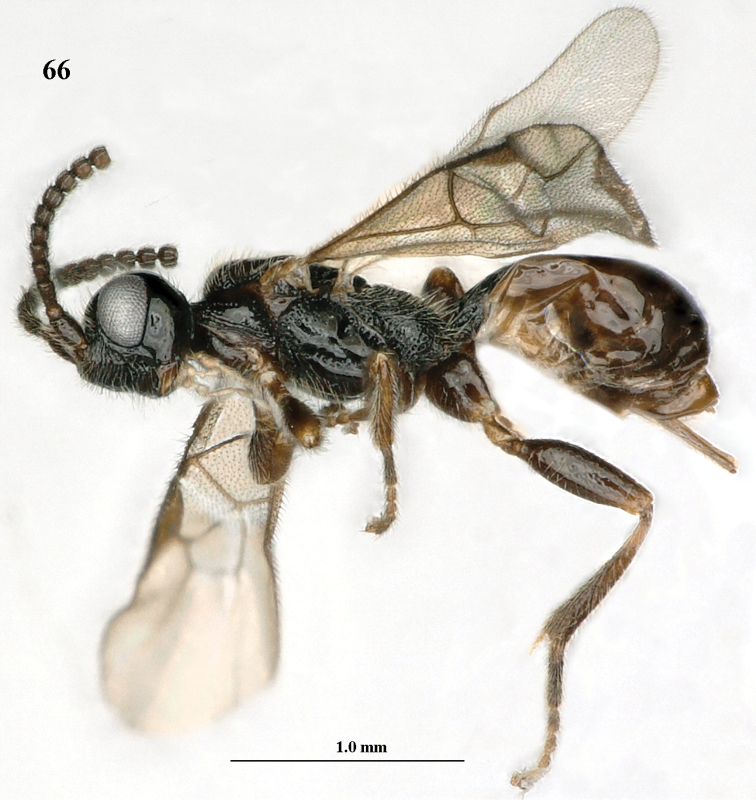
*Paramannokeraia juliae* gen. n. & sp. n., ♀, holotype, habitus, lateral aspect.

#### Diagnosis.

Antenna of ♀ with 19 robust segments, apical segments pedunculate (Fig. 72), of ♂ cylindrical and elongate (Fig. 81); head transverse, not enlarged behind eyes in dorsal view (Fig. 74); face mainly sparsely punctate (Fig. 73); mesosoma of ♀ normal, with mesoscutum distinctly above upper level of pronotum (Fig. 68); notauli on mesoscutal disk widened (Fig. 69); propodeum rather sparsely rugulose but sublaterally largely smooth (Fig. 69); base of hind coxa smooth dorsally (Fig. 68); hind femur rather swollen (Fig. 71); fore tibia rounded apically, without tooth-like protuberance apically (Fig. 76); fore and middle tarsi of ♀ widened (Figs 66, 76); basal half of antenna of both sexes and legs dark brown; setose part of ovipositor sheath about 0.4 times as long as hind tibia (Fig. 66); both sexes macropterous.

#### Description.

Holotype, ♀, length of fore wing 2.5 mm, and of body 2.7 mm.

*Head.* Antenna with 19 segments, length of third segment 1.1 times fourth segment, third, fourth and penultimate segments 1.6, 1.4 and 0.9 (without pedunculus 0.8) times as long as wide, respectively (Fig. 72) and with apical 12 segments pedunculate, medially antenna as wide as apically; length of maxillary palp 0.8 times height of head; occipital carina complete, low dorsally (Fig. 75); eye 1.5 times as long as temple in dorsal view; temples subparallel-sided behind eyes; OOL:diameter of posterior ocellus:POL = 9:5:11; frons smooth and shiny, with some long setae, convex, with shallow median groove, and anteriorly flattened; face with some rugae dorsally below antennal sockets and remainder sparsely coarsely punctate (Fig. 73); clypeus depressed and smooth ventrally, with ventral rim slightly upcurved, dorsally weakly convex and with some coarse punctures (Fig. 73); length of malar space 1.1 times basal width of mandible; occipital carina joining hypostomal carina and occipital flange subcircular; mandible depressed medially and shiny, apically with large upper and small lower tooth.

**Figures 67–76.**
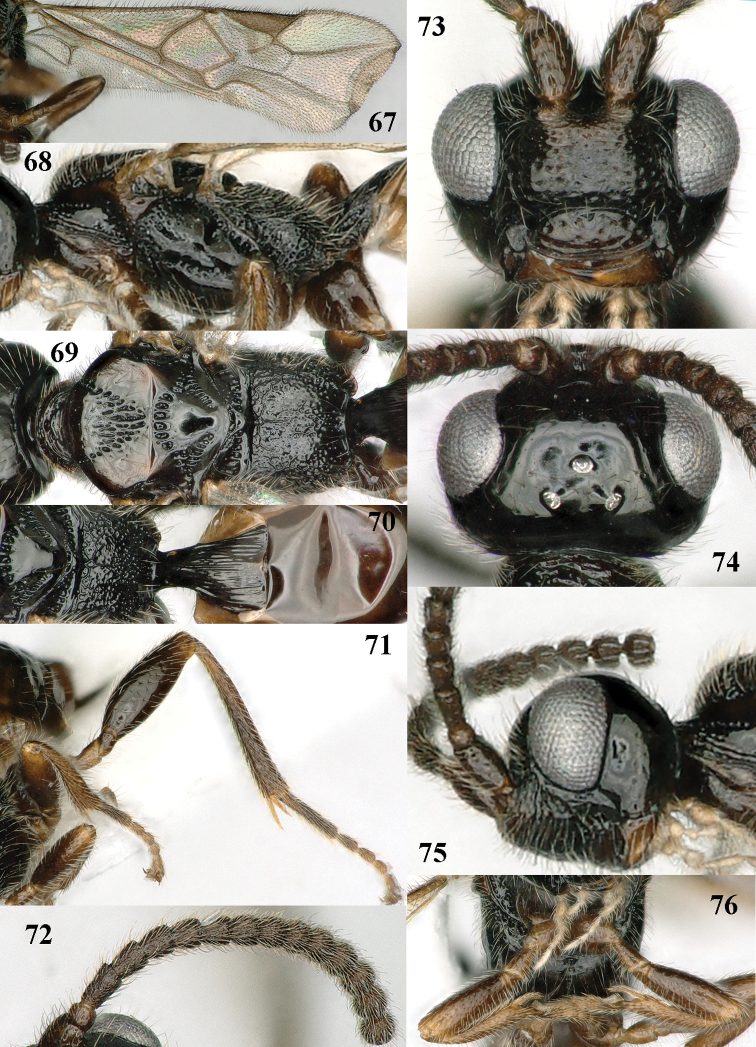
*Paramannokeraia juliae* gen. n. & sp. n., ♀, holotype. **67** wings **68** mesosoma, lateral aspect **69** mesosoma, dorsal aspect **70** propodeum, first–third metasomal tergites, dorsal aspect **71** hind leg, lateral aspect **72** antenna **73** head, anterior aspect **74** head, dorsal aspect **75** head, lateral aspect **76** fore legs, inner aspect.

*Mesosoma.* Length of mesosoma 1.9 times its height; dorsal pronope and antescutal depression absent; side of pronotum largely punctate-rugose ventrally, largely smooth medially, with narrow crenulate groove antero-dorsally and punctate-costate posteriorly; epicnemial area punctate dorsally; precoxal sulcus complete, narrowly crenulate-punctate (Fig. 68); remainder of mesopleuron smooth; mesosternal suture deep and coarsely crenulate; postpectal carina absent; notauli complete, coarsely punctate and rather wide, ending in wide punctate area (Fig. 69); scutellar sulcus with five costae; scutellum flat, smooth and shiny; metapleuron punctate medially and coarsely reticulate-punctate ventrally; postpectal carina absent and area between middle coxae with few punctures; propodeum rather sparsely rugulose but sublaterally largely smooth (Fig. 69), posterior face weakly differentiated and without an obtuse tubercle postero-laterally (Fig. 68).

*Wings.* Fore wing: 1-M weakly curved; 1-SR short (Fig. 67); marginal cell open anteriorly because of most of 1-R1 absent and sclerotized part of 1-R1 about 0.2 times as long as pterostigma (Fig. 67); vein r emitted distinctly after middle of pterostigma; r:3-SR:SR1 = 2:11:62; vein SR1 straight; 2-SR:3-SR:r-m = 27:11:15; 2-M much longer than 3-SR; m-cu slightly postfurcal; 1-CU1 oblique and narrow, about as long as cu-a; 1-CU1:2-CU1 = 5:27; basal and subbasal cells of fore wing setose as other cells. Hind wing: marginal cell subparallel-sided medially and obsolescent apically; M+CU:1-M:1r-m = 41:18:10.

*Legs.* Hind coxa basally smooth; tarsal claws with wide truncate lamelliform lobe (Fig. 71); length of femur, tibia and basitarsus of hind leg 3.0, 7.3 and 6.0 times as long as their maximum width; fore femur rather inflated, 2.9 times longer than wide and apically rounded (Fig. 76); fore and middle tarsi widened (Figs 66, 76).

*Metasoma.* First tergite 1.5 times longer than its apical width, distinctly petiolate (Fig. 70), with incomplete straight striae, dorsal carinae unite to form a median carina and dorsope deep and large (Fig. 70), only basal quarter closed ventrally; laterope absent, tergite widened latero-basally; second tergite smooth; ovipositor sheath somewhat widened and obtuse apically (Fig. 66), its setose part 0.14 times as long as fore wing and 0.40 times hind tibia; ovipositor with minute subapical nodus and widened basally.

*Colour.* Black; antenna, metasoma except black first tergite and legs dark brown, but hind trochanter and tibial spurs brown; tegulae and palpi pale yellowish; pterostigma and veins brown; wing membrane weakly infuscate.

**Figures 77–83.**
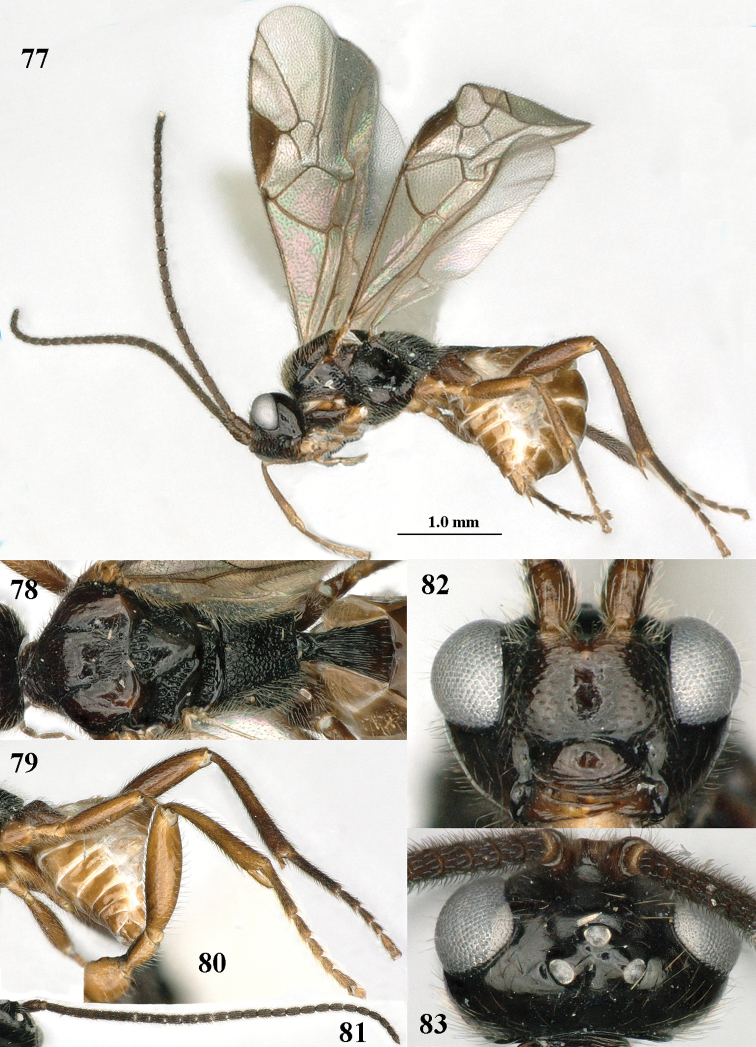
*Paramannokeraia juliae* gen. n. & sp. n., ♂, paratype. **77** habitus, lateral aspect **78** mesosoma, dorsal aspect **79** hind leg, lateral aspect **80** fore leg, lateral aspect **81** antenna **82** head, anterior aspect **83** head, dorsal aspect.

*Male*. Similar to female holotype except for the shape of the antennal segments, slender fore and middle tarsi (Figs 77, 80, the rugose area between middle coxae and the different sculpture of the propodeum and face (Figs 78, 82). Antenna with 28 segments, length of fore wing 3.6 mm, and of body 3.6 mm; face and clypeus rather finely punctate; metasoma (except most of first tergite) brown; mesoscutum less flattened; medio-posterior punctate area of mesoscutum small; propodeum largely finely rugulose; first tergite 1.5 times longer than wide apically and distinctly longitudinally striate.

#### Etymology.

Named after Dr Julia Stigenberg (Stockholm), who generously made the holotype available for this study.

#### Distribution.

Australia (Tasmania). Collected in January–March.

### Planitorus

Taxon classificationAnimaliaHymenopteraBraconidae

van Achterberg, 1995

Figs 84–96

Planitorus van Achterberg, 1995: 46–47.

#### Type species.

*Planitorus breviflagellaris* van Achterberg, 1995 (examined).

#### Diagnosis.

**Figures 84–96.**
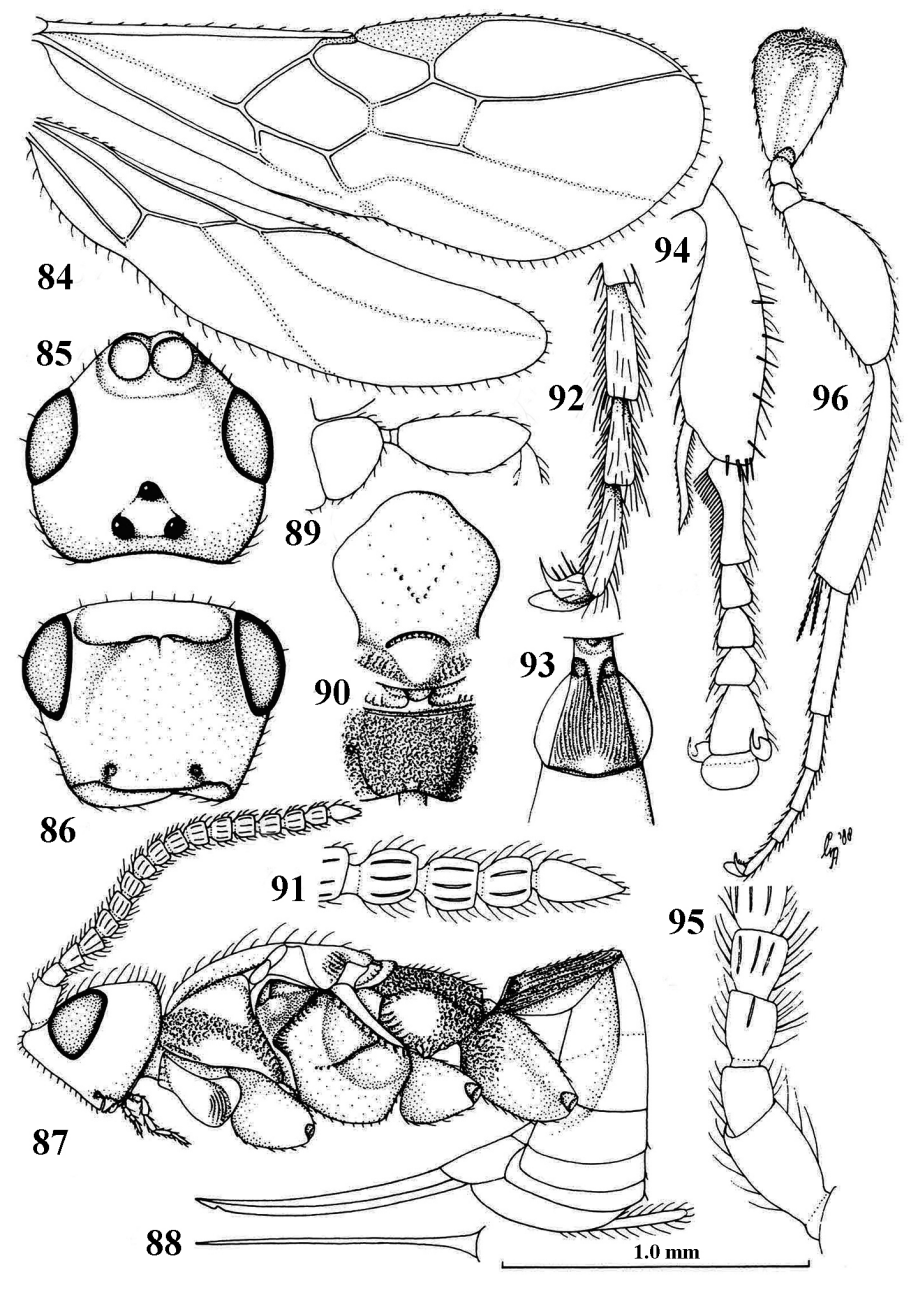
*Planitorus breviflagellaris* van Achterberg, ♀, holotype. **84** wings **85** head, dorsal aspect **86** head, frontal aspect **87** habitus, lateral aspect **88** ovipositor, ventral aspect **89** fore femur, lateral aspect **90** mesosoma, dorsal aspect **91** apex of antenna **92** outer hind claw, lateral aspect **93** first metasomal tergite, dorsal aspect **94** fore tibia and tarsus, mainly lateral aspect **95** base of antenna, lateral aspect **96** hind leg, lateral aspect. 84, 87–90, 93, 95: 1.0× scale-line; 85, 86: 1.6×; 91, 92, 94, 96: 2.5×. From: van Achterberg (1995).

Antenna of ♀ with 17 segments, and segments of apical half moniliform (Figs 87, 91), of ♂ unknown; antennal sockets touching each other (Fig. 85); clypeus not differentiated from face (Fig. 86); head elongated below eyes (Fig. 86); pronotal collar below level of mesoscutum (Fig. 87), but mesoscutum low anteriorly; notauli largely reduced, united posteriorly (Fig. 90); scutellar sulcus narrow and curved (Fig. 90); mesosternal sulcus absent and area smooth; vein M+CU1 of fore wing of type species largely unsclerotized (Fig. 84); fore tibia with spiny setae (Fig. 94); hind coxa only basally rugose (Fig. 87); fore and middle tarsal claws with a lamelliform lobe (Fig. 94) and hind claws simple (Fig. 92); dorsope of first tergite distinct and tergite distinctly widened posteriorly (Fig. 93); ovipositor strongly compressed (Figs 87, 88); ♀ macropterous.

#### Distribution.

Australia: one species.

#### Biology.

Unknown.

### Planitorus breviflagellaris

Taxon classificationAnimaliaHymenopteraBraconidae

van Achterberg, 1995

Figs 84–96

Planitorus breviflagellaris van Achterberg, 1995: 47-48, 192.

#### Diagnosis.

See generic diagnosis.

#### Distribution.

Australia (Queensland, A.C.T.). Collected in December–March.

#### Notes.

A headless and also otherwise severely damaged male from near Mount Barker (HIC: Western Australia, 25.iv.2000, DNA voucher BJS101 (as “*Planitorus* sp.” in Sharanowski et al. (2011)), and incorrectly labelled as “Mount Baker”) may belong to an undescribed second species. It has the notauli entirely absent, vein M+CU1 of fore wing largely sclerotized and the precoxal sulcus present medially.

## Supplementary Material

XML Treatment for Planitorini

XML Treatment for Mannokeraia

XML Treatment for Mannokeraia albipalpis

XML Treatment for Mannokeraia aptera

XML Treatment for Mannokeraia nigrita

XML Treatment for Mannokeraia punctata

XML Treatment for Paramannokeraia

XML Treatment for Paramannokeraia gibsoni

XML Treatment for Paramannokeraia juliae

XML Treatment for Planitorus

XML Treatment for Planitorus breviflagellaris
